# Outcome of non cardiac surgical patients admitted to a multidisciplinary indian ICU

**DOI:** 10.1186/2197-425X-3-S1-A737

**Published:** 2015-10-01

**Authors:** HG Pradeep Kumar, MS Kalaiselvan, MK Renuka, AS Arunkumar

**Affiliations:** Sri Ramachandra Medical College and Research Institute, Dept of Critical Care Medicine, Chennai, India; Sri Ramachandra Medical College and Research Institute, Dept of Anaesthesiology, Chennai, India

## Introduction

Perioperative complications after non cardiac surgery are relatively frequent and potentially dangerous[1, 2]. Data is also limited in these group of patient population in India. Hence we planned to study the profile of perioperative patients.

## Objective

Study the clinical profile and outcome of perioperative patients admitted to a multidisciplinary ICU and identify risk factors if any for post operative mortality.

## Methods

This is a prospective observational study on all perioperative patients admitted to a multidisciplinary ICU. The study was conducted between MAY 2014-NOV 2014. The primary outcome analyzed was perioperative complications and hospital mortality. The secondary outcome analyzed were duration of ICU stay, ventilator free days and ICU free days. We expressed results as mean ± standard deviation, and frequencies for qualitative variables. We used the Fisher exact test and the Mann-Whitney's test with a significance level of 0.05.

## Results

This study included 185 patients of which 55%(n=102) were male. Mean age was 49.8 ± 18.3 years, with 34%(n=63) above 65 years. Mean admission APACHE-II score was 11 ± 6.38 and SOFA score was 3.16 ± 2. The mean discharge SOFA score was 2.51 ± 2.4.60%(n=109) of patients underwent elective surgery while 41%(n=76) had emergency surgery. The most common reason for post operative ICU admission was for elective mechanical ventilation in view of co-existing medical conditions 47%(n=87). The mean duration of surgery was 171.6 ± 97.8 mins. The most common surgical procedures were gastro-intestinal 30% (n=56) followed by orthopaedic 20.5%(n=38) and genito-urinary 16%(n=16%). 66.5%(n=123) patients received a general anesthetic alone of whom 67%(n=83) required elective mechanical ventilation in ICU. The overall mortality was 7.6%(n =14). Post operative surgical complications were seen in 2.2%(n=4) and nonsurgical complications were seen in 14%(n=26)patients of which AKI was most common 38.5%(n=10 of 26). Secondary outcome measures were (Mean ± SD) ICU LOS 3.05 ± 2.1 days, ICU free days6.28 ± 3.6 days and Ventilator free days 6.59 ± 4.1 days. The discharge SOFA (2.37 ± 2.2 vs 4.21 ± 4.2), admission APACHE-II (11.17 ± 6.5 vs 20.14 ± 4.0) and ICU LOS(2.96 ± 2.0 vs 4.14 ± 3.3) were significantly higher in non-survivors on univariate analysis(p < 0.05). The need for Post-operative fluid resuscitation, poor glycaemic control, presence of coagulopathy and surgical complications were also associated with higher hospital mortality (p < 0.05).

## Conclusions

Non cardiac surgical patients requiring ICU admission has significant hospital mortality.
